# Green Synthesis of MnO Nanoparticles Using *Abutilon indicum* Leaf Extract for Biological, Photocatalytic, and Adsorption Activities

**DOI:** 10.3390/biom10050785

**Published:** 2020-05-19

**Authors:** Shakeel Ahmad Khan, Sammia Shahid, Basma Shahid, Urooj Fatima, Saddam Akber Abbasi

**Affiliations:** 1 Center of Super-Diamond and Advanced Films (COSDAF), Department of Chemistry, City University of Hong Kong, 83 Tat Chee Avenue, Kowloon, Hong Kong, China; 2 Department of Chemistry, University of Management and Technology, Lahore-54770, Pakistan; sammia.shahid@umt.edu.pk (S.S.); basmashahidrps@gmail.com (B.S.); urooj.fatima@umt.edu.pk (U.F.); 3 Department of Mathematics, Statistics, and Physics, Qatar University, Doha 2713, Qatar; sabbasi@qu.edu.qa

**Keywords:** green synthesis, plant extract, biological activities, photocatalysis, adsorption

## Abstract

We report the synthesis of MnO nanoparticles (AI-MnO NAPs) using biological molecules of *Abutilon indicum* leaf extract. Further, they were evaluated for antibacterial and cytotoxicity activity against different pathogenic microbes (*Escherichia coli*, *Bordetella bronchiseptica*, *Staphylococcus aureus*, and *Bacillus subtilis*) and HeLa cancerous cells. Synthesized NAPs were also investigated for photocatalytic dye degradation potential against methylene blue (MB), and adsorption activity against Cr(VI) was also determined. Results from Scanning electron microscope (SEM), X-ray powder diffraction (XRD), Energy-dispersive X-ray (EDX), and Fourier-transform infrared spectroscopy (FTIR) confirmed the successful synthesis of NAPs with spherical morphology and crystalline nature. Biological activity results demonstrated that synthesized AI-MnO NAPs exhibited significant antibacterial and cytotoxicity propensities against pathogenic microbes and cancerous cells, respectively, compared with plant extract. Moreover, synthesized AI-MnO NAPs demonstrated the comparable biological activities results to standard drugs. These excellent biological activities results are attributed to the existence of the plant’s biological molecules on their surfaces and small particle size (synergetic effect). Synthesized NAPs displayed better MB-photocatalyzing properties under sunlight than an ultraviolet lamp. The Cr(VI) adsorption result showed that synthesized NAPs efficiently adsorbed more Cr(VI) at higher acidic pH than at basic pH. Hence, the current findings suggest that *Abutilon indicum* is a valuable source for tailoring the potential of NAPs toward various enhanced biological, photocatalytic, and adsorption activities. Consequently, the plant’s biological molecule-mediated synthesized AI-MnO NAPs could be excellent contenders for future therapeutic applications.

## 1. Introduction

Bacterial infections are still a major cause of fatalities over the globe. The rapid emergence of resistance to multiple drugs in different bacteria have further made the condition more problematic. It has been estimated that about 50% of hospitalized patients are infected by multiple drug-resistant bacteria over the globe every year [1]. Therefore, it is necessary to develop new alternatives to combat such drug-resistant bacteria. In addition to microbial diseases, cancer is also considered a major cause of fatalities in humans. It is estimated that from 1975 to 2000, the number of cancer patients has doubled [2]. Many advances have taken place in the area of molecular and cellular biology for improving the treatment of cancerous cells by chemotherapy. However, chemotherapy treatments also have adverse effects besides their positive norms, so it is necessary to develop new anticancer drugs with enhanced biocompatibility, efficacy, and reduced adverse impacts.

Moreover, irrespective of biological diseases, human beings, plants, and animals are also getting affected by the adverse impacts of the bad surrounding environment. Our environment is being polluted due to human-made pollution produced by different industrial sectors, including plastic, rubber, paper, textile, and leather. All these industrial sectors extensively use different kinds of synthetic dyes, including methylene blue, Congo red, acid black 1, acid black 234, and acid black 210 [3,4]. All these dyes are carcinogenic beyond their permitted limit. If these dyes are discharged continuously without proper treatment or conversion into less harmful products, they can cause serious hazardous effects on the environment and ultimately on all living species. Therefore, the development of simple, robust, efficient methods based on a photocatalyst system has attained enormous attention to disintegrate such synthetic dyes into less-toxic chemical waste.

In this instance, metal (Ag, Au, Pt, Cu, Zn, etc.) and their metal oxide (MgO, NiO, CuO, ZnO, TiO_2_, etc.) nanoparticles (NAPs) are considered as the most viable opportunity due to their unique physical characteristics, including the large surface area to volume ratio, controlled morphology (uniform and homogeneous), smaller size, and light-absorbing properties [5,6,7,8,9,10,11,12,13,14]. These features enable them to be used widely in different applications, including biomedicine, biosensing, and optoelectronics. Metal and metal oxide NAPs have been widely exploited for their applications in biomedicine, including antibacterial, antimycotic, anticancer, antilarvicidal, and antidiabetic [11,12,13,14]. However, MnO NAPs have gained importance in the synthesis and manufacturing processes because of their lower toxicity [15]. Mostly, NAPs are synthesized using chemical and physical techniques. Both techniques demand high energy, toxic chemicals for reduction and capping, and are not easily scalable. A most important factor, these techniques compromise the biocompatibility of NAPs due to the use of toxic chemicals in the synthesis process because these chemicals are left behind on the NAPs’ surface even after washing several times. Hence, their usages in biomedical applications are compromised [16,17]. Therefore, the synthesis of NAPs using biological techniques, especially plant-based, is an alternative approach to these conventional methods. Plant-based biological techniques have several advantages, including one-pot synthesis, robust, economical, eco-friendly, and furthermore, they use the plant’s biological molecules as a reducing and capping agent. The plant-based biological techniques also reduce the chances of NAPs’ biocompatibility being compromised due to the involvement of plant’s biological molecules (proteins, macromolecule carbohydrates, glycoproteins, proteoglycans, biological polyacids, and nucleic acids) in the synthesis process [8,9,10] and use of these macromolecules is considered as safe and environmentally friendly.

In this work, we have synthesized MnO NAPs for the first time to the authors’ knowledge using biological molecules of leaf extract of *Abutilon indicum*. *Abutilon indicum* is a medicinal plant that is widely employed for the treatment of several diseases such as jaundice, gout, asthma, tuberculosis, toothache, swelling of the bladder, coughs, ulcers, diarrhea, and chest infection. *Abutilon indicum* has several biologically active phytomacromolecules such as alkaloids, flavonoids, steroids, tannins, terpenoids, and saponins [18] which demonstrate hepatoprotective [19], antioxidant [20], anti-inflammatory [21], antimicrobial [22], hypoglycemia [23], antidiarrheal [24], antiproliferative [25], anti-arrhythmic [26], antilarvicidal [27], anticancer [28], anti-asthmatic [29], antidiabetic [30], anticonvulsant [31], and analgesic activities [32]. Thus, in the present research, AI-MnO NAPs were synthesized using *A. indicum* phytomolecules as a reducing and stabilizing agent. The green-synthesized AI-MnO NAPs were further evaluated for their different applications, i.e., anticancer, antibacterial, photocatalytic against methylene blue, and removal of heavy metal ion (CrVI) by adsorption.

## 2. Materials and Methods

### 2.1. Chemicals

All the chemicals (MnSO_4_·H_2_O, K_2_Cr_2_O_7_, HCl, NaOH, DMSO, methylene blue dye, nutrient agar, nutrient broth, ethanol, and methanol) of analytic grade were purcased from Sigma-Aldrich Darmstadt, Germany.

### 2.2. Collection of Plant Material

Fresh *Abutilon indicum* leaves were collected and identified by Dr. Zaheer-u-din Khan (Department of Botany, GC University, Pakistan). The voucher specimen was deposited in the herbarium under number GC. Herb. Bot. 68. The plant was dried for about two to three weeks in a shady area. These leaves were then ground into a powder and used for extraction.

### 2.3. Plant Extract Preparation and Synthesis of AI-MnO NAPs

**Figure 1:**
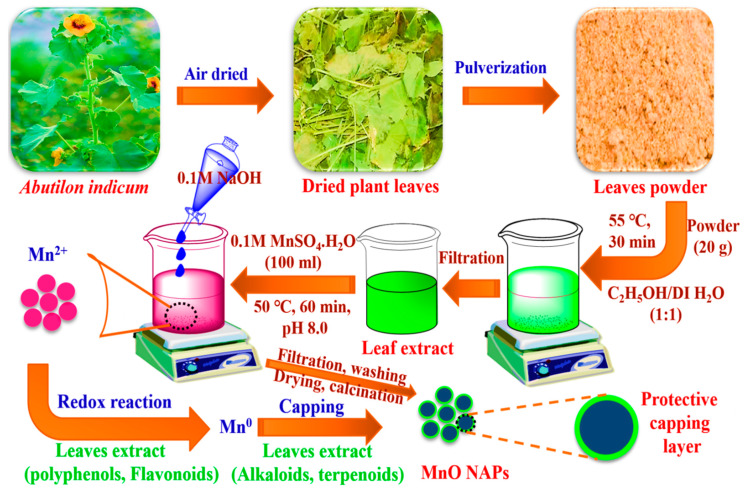
Schematic presentation for the green synthesis of AI-MnO nanoparticles (NAPs) using biological molecules of *Abutilon indicum* leaf extract.

The preparation of plant extract and synthesis of AI-MnO NAPs were carried out following the procedure reported by [33] with slight modifications. Accordingly, the 20-g of leaves powder of *A. indicum* was added to a 500-mL beaker, then methanol/deionized water (1:1 ratio) with a 150-mL volume of each was transferred for extraction (Figure 1). It was placed on a magnetic hot plate and underwent stirring for about 30 min at 55 °C and allowed to settle overnight. Then, it was filtered with filter paper to get the plant extract. After that, 100 mL of 0.1 M MnSO_4_·H_2_O was taken in a 500-mL beaker, and 100 mL of plant extract was added to it. A total of 0.1-M NaOH solution was added dropwise to the beaker with constant stirring for about 1 h at pH 8.0 and 50 °C (Figure 1). Afterwards, they were filtered and washed with hexane or ethanol to remove impurities. Then, obtained precipitates were dried in an oven at 90 °C for 1 h and put in a muffle furnace for calcination at 150 °C for 2 h. They were then used for further characterization.

### 2.4. Characterization

The green-synthesized AI-MnO NAPs were then characterized by employing different spectroscopic techniques. X-ray diffraction crystallography (XRD) was used for determining the crystalline structure of the fabricated NAPs. Energy-dispersive X-ray spectroscopy (EDX) analysis was performed to verify the chemical composition of the green-synthesized AI-MnO NAPs. Morphology determination of the synthesized NAPs was carried out by using a scanning electron microscope (SEM). Fourier transform infrared (FTIR) analysis of the synthesized NAPs was executed to determine whether biological molecules of plant’s leaves extract are involved or not. The photocatalytic and adsorption activities were performed against methylene blue (MB) dye and Cr(VI), respectively, using a UV–visible spectrophotometer.

### 2.5. Antibacterial Activity

Antibacterial activity of the synthesized AI-MnO NAPs was evaluated on bacterial species, which include *Escherichia coli*, *Bordetella bronchiseptica*, *Staphylococcus aureus*, and *Bacillus subtilis* by using the disk-diffusion agar method [34]. Leflox (standard antibacterial drug) and DMSO at 0.001 g/mL were used as a positive and negative control, respectively. Different concentrations of the green-synthesized AI-MnO NAPs were prepared in DMSO—i.e., 10 µg/mL, 20 µg/mL, 30 µg/mL, and 40 µg/mL—and placed in a sonicator at 36 °C for 15 min to ensure uniform dispersion. A total of 30 mL of molten nutrient agar was added in sterile Petri dishes, and 3 mL of inoculum was inoculated in it. At four peripheral positions, holes were made and filled with the reference standards and sample dilutions. After 24 h of incubation at 37 °C, a clear zone was formed around disks. The diameter of the inhibition zones was measured using a Vernier caliper.

### 2.6. Cytotoxicity Activity

HeLa cancer cell lines were used to assess the cytotoxicity activity of synthesized AI-MnO NAPs using MTT colorimetric assay [35]. The HeLa cancer cells were kept in Dulbecco’s Modified Eagle’s Medium (DMEM) supplied with streptomycin (100 μg/mL), penicillin (100 U/mL), and 10% FBS (fetal bovine serum) in a humidified atmosphere comprising of CO_2_ (5%) and air (95%) at 37 °C. The HeLa cancer cells were cultured in 100 μL of DMEM in a 96-microtiter plate for 24 h at 37 °C in 5% CO_2_ to get cell confluency up to 5 × 10^4^ cells/well. The cultured cancer cells were further incubated for 24 h at 37 °C with different concentrations (1, 5, 10, 15, 30, 60, 120 μg/mL) of green-synthesized AI-MnO NAPs. Cells treated with the standard drug (doxorubicin) were termed as positive control and cells without any treatment as negative control. Afterwards, cancer cells were centrifuged to remove the supernatant and subsequently washed using the phosphate buffer saline (PBS) solution. A total 10 μL of MTT labeling agent at the concentration of 0.5 mg/mL was then added to each well; the 96-microtiter plate was again incubated for 4 h at 37 °C in a humidified atmosphere comprising of CO_2_ (5%) and air (95%); and subsequently, 100 μL of DMSO was added to each well to solubilize the undissolved crystals of formazan, then kept in a shaker for about 10 to 15 min. The absorption maxima of the formazan in each well were determined using a Varian Eclipse spectrophotometer at 570 nm with reference at 655 nm. The percentage of cell viability can be calculated using the given formula [35]:

Percentage of cell viability = (OD value of treated cells)/(OD value of negative control) × 100.

### 2.7. Biocompatibility Analysis

The biocompatibility of the green-synthesized AI-MnO NAPs was determined following the standard protocol reported by Khan et al. [36].

### 2.8. Photocatalytic Activity Against Methylene Blue

The photocatalytic degradation activity of green-synthesized AI-MnO NAPs was determined using methylene blue dye as a model system [3]. A total of 0.05 g of green-synthesized AI-MnO NAPs were added to 100 mL of methylene blue dye solution (0.1%, *w*/*v*), and this suspension was further kept in the dark for 1 h to reach adsorption–desorption equilibrium. Methylene blue solution without any treatment served as control. The resultant suspensions were then irradiated with the UV lamp (254–370 nm, Osram ultra-vitalux, 300 W, Munich, Germany) and solar light (80–90 Klux, LT300, Extech, Leeds, UK) without any external pressure and changing pH. The distance between suspensions surface and UV lamp was 12 cm. The temperature of the samples under the sunlight and UV lamp was 30 °C. During the whole irradiation process, the suspension was stirred continuously, employing a magnetic stirrer for uniform mixing. A 5-mL aliquot was then sampled out for initial concentration. About 5 mL was further sampled in regular intervals of time—i.e., after every 30 min for 3 h—and centrifuged to separate the photocatalyst (AI-MnO NAPs). The suspension was then studied using a UV spectrophotometer at the specific absorption peak (665 nm) of methylene blue to determine the remaining dye contents. The percentage degradation of the dye with a UV lamp and solar light was calculated using the following formula:

% D = (C_o_ − C_t_)/C_o_ × 100,

where C_o_ is the initial dye concentration and C_t_ is the dye concentration at time t (min).

Three successive runs in sunlight irradiation were conducted to examine the recovery and stability of AI-MnO NAPs photocatalyst. Then, after each run, AI-MnO NAPs photocatalyst was removed, washed with deionized water and ethanol, dried, and the same NAPs reused. After that, their degradation efficiency was investigated.

### 2.9. Cr(VI) Adsorption Capacity of Synthesized AI-MnO NAPs

The green-synthesized AI-MnO NAPs were investigated for adsorption activity against Cr(VI) ions in a glass flask under magnetic stirring with constant speed [37]. In brief, 20 mg of potassium dichromate as Cr(VI) source was dissolved in 100 mL of deionized water. Different amounts (0.01, 0.02, 0.03, 0.04, and 0.05 g) of adsorbent (AI-MnO NAPs) were then mixed separately with 100 mL of Cr(VI) solution at pH 4.50, 7, and 9 at 30 °C. A 5-mL aliquot was sampled after every 15 min and centrifuged for removing adsorbent from solution. The remaining Cr(VI) ions were determined using a UV spectrophotometer at their specific peaks. The adsorption capacity (R) (mg/g) was calculated using the following formula:

R = (C_o_ − C_t_)/m × V,

where C_o_ is the initial concentration of Cr(VI), C_t_ is the concentration of Cr(VI) at time t, m is the adsorbent mass, and v is the solution volume.

## 3. Results and Discussion

### 3.1. XRD Analysis

**Figure 2:**
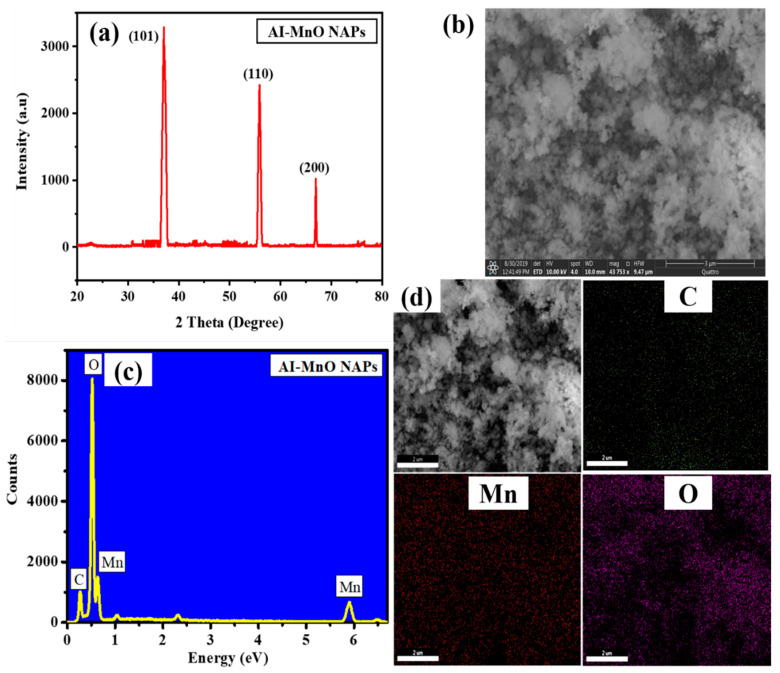
(**a**) X-ray powder diffraction (XRD) spectra, (**b**) scanning electronic miscroscope (SEM), (**c**) Energy-dispersive X-ray (EDX), and (**d**) EDX apping of green-synthesized AI-MnO NAPs using leaf extract of *Abutilon indicum*.

Figure 2a displayed the XRD pattern of green-synthesized AI-MnO NAPs. The XRD pattern results demonstrated the different diffraction peaks, which agree with JCPDS no. 78-0424 as indexing to (101), (110), and (200) crystal planes at an angle of 37°, 56°, and 67°, respectively [38]. The highest peak intensity was shown at the Miller index (101) at an angle of 37°. The higher peak intensity and their sharpness further corroborated that the synthesized NAPs are highly crystalline. Moreover, it has also been perceived from the XRD pattern that no peak associated with impurity was detected, which displayed that the synthesized AI-MnO NAPs are pure.

### 3.2. SEM Studies

**Figure 3:**
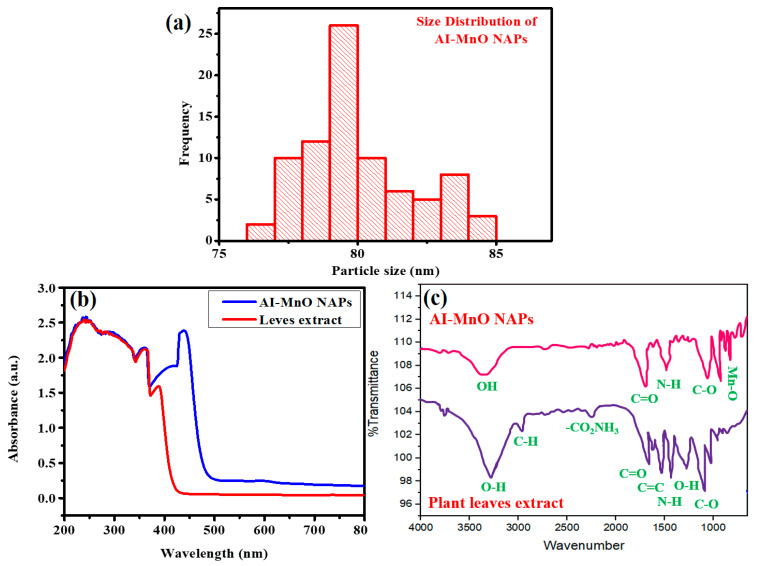
(**a**) The particle-size distribution of AI-MnO NAPs. (**b**) ultraviolet (UV)–visible and (**c**) Fourier-transform infrared spectroscopy (FTIR) spectra of the green-synthesized AI-MnO NAPs and leaf extract.

SEM was used for identifying the morphology and size of NAPs. Figure 2b shows the SEM image of the green-synthesized AI-MnO NAPs. The SEM image of the green-synthesized AI-MnO NAPs revealed that they have spherical morphology. The SEM image further showed that the synthesized AI-MnO NAPs were uniformly distributed. An average size of 80 ± 0.5 nm was observed for the synthesized AI-MnO NAPs investigated by particle size distribution analysis. Figure 3a indicates that the particle size distribution of AI-MnO NAPs is right-skewed.

### 3.3. EDX Analysis

The EDX analysis was carried out to examine the formation and chemical composition of the green-synthesized AI-MnO NAPs. Figure 2c,d displays the EDX pattern and EDX mapping, respectively, and these results were validated with the successful green synthesis of the NAPs using biological molecules of leaf extract of *A. indicum*. The presence of chemical element (Mn) in the synthesized NAPs was validated by its EDX peaks at 0.63 keV and 5.91 keV in the EDX spectrum [39]. In addition to Mn EDX peaks, the peaks corresponding to carbon (C) and oxygen (O) also clearly appeared in the EDX spectrum, which corroborated the adsorption of biological molecules on NAPs surface from the plant extract. The EDX results were further affirmed that the NAPs were free from impurity. Hence, it is apparent from the EDX results that NAPs of our interest have been successfully synthesized using plant leaf extract. EDX pattern results of green-synthesized AI-MnO NAPs were in agreement with the previously reported literature [39].

### 3.4. UV-Visible and FTIR Analysis

The UV-visible and FTIR analyses of plant extract and green-synthesized AI-MnO NAPs were further carried out to determine the absorption maxima and different functional groups of biological molecules of *A. indicum* leaf extract, respectively, that are involved in reduction and capping. The UV–visible analysis demonstrated that plant leaf extract displayed absorption maxima in the UV region (200–390 nm) which corroborated that the leaf extract is a rich source of polyphenols and flavonoids as these biological phytomolecules absorb UV light due to the presence of hydroxyl (OH) moieties [40,41]. Green-synthesized AI-MnO NAPs presented a broad absorption band with two characteristic absorption peaks at 380 nm (biological phytomolecules) and 460 nm (Mn-O) (Figure 3b). The slight redshift in the absorption maxima of Mn-O was observed compared to reported works [42,43]. This might be attributed to the fact that the donation of non-bonding electrons from phytomolecules to the vacant *d*-orbital of Mn facilitated the electron transition, which shifted the absorption maxima to the higher wavelength [44].

The FTIR analysis demonstrated that plant leaf extract has biological phytomolecules with different molecular functionalities—such as O–H, C–H, CO_2_NH_3_, C=O, C=C, N–H, C–O (Figure 3c)—and these FTIR signals were found in close agreement with the previous literature [40,41]. On the other hand, green-synthesized AI-MnO NAPs not only presented the characteristic FTIR signal for Mn–O at about 580 cm^–1^, but also showed other FTIR signals for O–H, C=O, N–H, C–O. The FTIR signal of Mn-O was consistent with the previous report [42,43]. Hence, these observations demonstrated that green-synthesized AI-MnO NAPs are entirely capped with the biologically active phytomolecules of plant leaf extract having such functional groups.

### 3.5. Synthesis Mechanism

Literature demonstrated that leaf extract of *A*. *indicum* possesses numerous biologically active phytochemical compounds such as lignin, volatile oils, fixed oils, carbohydrates, steroids, terpenoids, tannins, saponins, flavonoids, phenolics, alkaloids, proteins anthraquinones, cardiac glycosides, etc. [40,41]. The presence of these phytomolecules in plant leaf extract and green-synthesized NAPs was further evident from the UV–visible and FTIR results. Therefore, because of the existence of these phytomolecules in leaf extract, they might be acting as reducing and capping agents during the synthesis of MnO NAPs. During synthesis, phytomolecules (flavonoids, phenolics, carbohydrates, etc.) reduced the Mn^+^ into their zero-valent species Mn^0^ by proving electrons through a redox reaction [45]. Afterwards, other phytomolecules such as alkaloids, proteins, surfactants, etc., simultaneously capped Mn^0^ zero-valent species to stabilize them (Figure 1). A similar mechanism of NAPs synthesis using plant leaf extract was also reported by [45].

### 3.6. Antibacterial Activity

**Figure 4:**
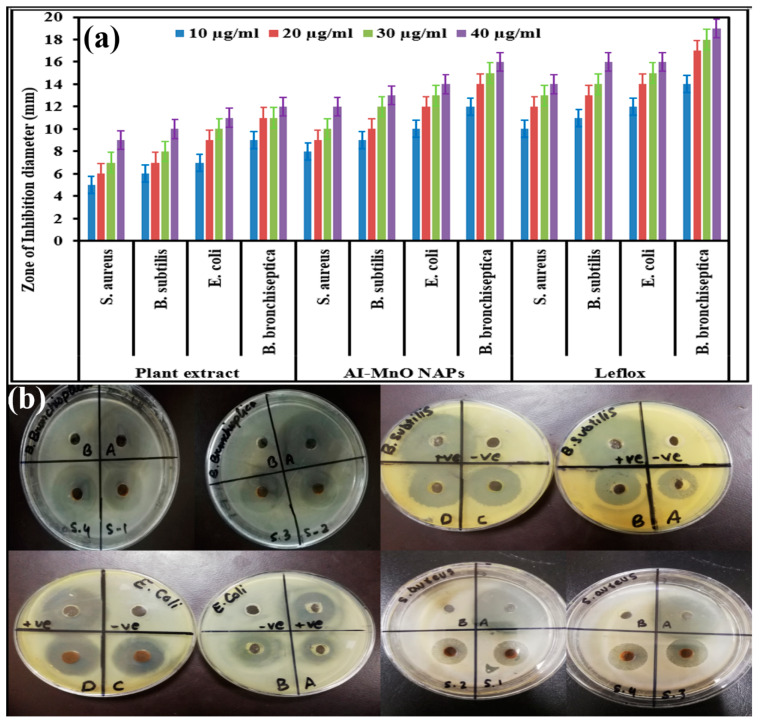
(**a**) The antibacterial activity of different concentrations of plant extract and green-synthesized AI-MnO NAPs in comparison to the standard drug (*F*-value = 1304.93, *p* < 0.05). (**b**) Zones of inhibition of green-synthesized AI-MnO NAPs against *B. bronchiseptica*, *E. coli*, *B. subtilis*, and *S. aureus*.

The antibacterial potential of green-synthesized AI-MnO NAPs was assessed following the disk-diffusion method against gram-positive and gram-negative bacteria in comparison to plant extract and standard antibacterial drug (Leflox). The results demonstrated that the highest antibacterial propensity in terms of zone of inhibition (ZOIs) was presented of course by the antibacterial drug in all its tested concentrations (10 µg/mL, 20 µg/mL, 30 µg/mL, 40 µg/mL) (Figure 4a). Meanwhile, the green-synthesized AI-MnO NAPs also displayed higher and comparable antibacterial activity to the standard antibiotic drug (Figure 4a,b), and the least antibacterial effect was observed with the plant extract. However, it is interesting to note that plant leaf extract itself was found to be biologically active against all the tested pathogenic bacterial strains. Moreover, the green-synthesized AI-MnO NAPs and plant extract were also found to exhibit concentration-dependent bactericidal activity against all the tested bacteria. The concentration of 10 µg/mL of the synthesized AI-MnO NAPs and plant leaf extract demonstrated the antibacterial activity in terms of ZOIs (5 ± 0.01 mm, 6 ± 0.03 mm, 7 ± 0.02 mm, and 9 ± 0.06 mm) and (8 ± 0.02 mm, 9 ± 0.04 mm, 10 ± 0.07 mm, and 12 ± 0.03 mm) against *S*. *aureus*, *B. subtilis*, *E. coli*, and *B. bronchiseptica*, respectively. With the increasing concentration of synthesized NAPs and plant extract from 10 µg/mL to 40 µg/mL, an increase in antibacterial activity in terms of ZOIs was observed against all the tested gram-positive bacteria and gram-negative bacterial strains. The same concentration-dependent antibacterial activity was reported in the previous literature [46,47]. However, it has been observed that the highest zone of inhibition was observed in gram-negative bacteria (*B. bronchiseptica* and *E. coli*) followed by gram-positive bacteria (*B. subtilis* and *S. aureus*) [46]. The good antibacterial results of the synthesized NAPs might be attributed to the NAPs’ physical characteristics (size, morphology, surface area) and their functionalization with the biologically active phytomolecules of plant leaf extract, because plant extract itself also exhibited good bactericidal performance [40,41]. Our green-synthesized AI-MnO NAPs were found to have good antibacterial performance in comparison to those of Arasu et al. which were synthesized by using aqueous extract of *Acorus* calamus rhizome [48].

**Figure 5:**
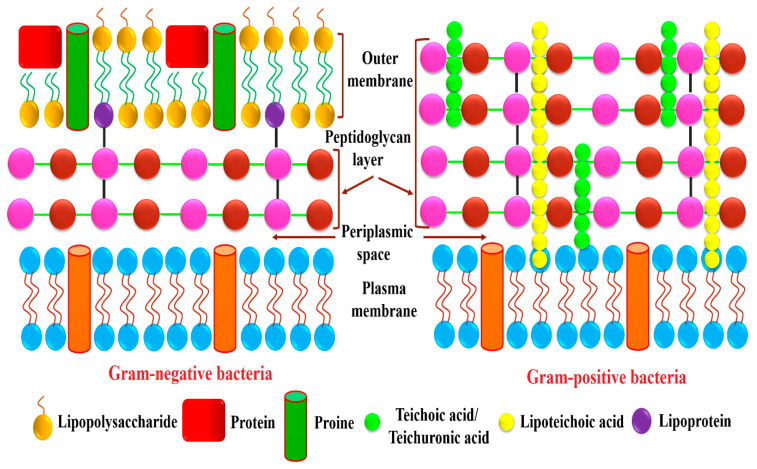
The cell-wall comparison between gram-positive and gram-negative bacterial strains.

Antibacterial results demonstrate that gram-negative bacteria are found to be more susceptible, and their growth was inhibited more strongly upon treatment with the green-synthesized AI-MnO NAPs than gram-positive bacteria. The same trend of growth inhibition was observed with the treatment of plant extract and standard drug. This is attributed to the differences found in the structure and composition of their cell walls [13,46,47]. The literature shows that a thicker layer of peptidoglycan is present in gram-positive bacterial cell wall along with covalently attached teichuronic and teichoic acid; while it is a thinner layer with an extra outer covering layer of lipopolysaccharides (periplasm) in gram-negative bacteria (Figure 5) [49]. Therefore, gram-negative bacteria having thin peptidoglycan layer are more vulnerable to NAPs than gram-positive bacterial strains. Further, the presence of negatively charged lipopolysaccharide biomolecules coatings is also the causative factor for the gram-negative bacteria being more sensitive to NAPs because they have a significant affinity for NAPs with positive surface charge. As a result, the NAPs are more effective against gram-negative than gram-positive bacteria [49].

The exact antibacterial mode of action of MnO NAPs against gram-positive and gram-negative bacteria is still unclear. However, much literature has proposed the possible mechanism of action of NAPs. Accordingly, MnO NAPs come into the vicinity of the negatively charged cell membrane of bacterial strains because of electrostatic attraction between them [46,48]. Finally, they bind to the cell membrane, which leads the cell membrane from a well-ordered to a disordered state. As a result of this alteration, the cell membrane loses its permeability and causes the leakage of bacterial cell electrolytes, which further destroys the structure and functioning capacity of mesosomes [13]. The NAPs further react with the thiol (–SH) functionality present in the cytosol, inhibit protein synthesis, and suppress the enzyme’s activity [50]. Such types of interference of NAPs with the different cellular organelles disrupt the intracellular cell-signaling process and decrease ATP synthesis in the cell powerhouse (mitochondria), which further enhances reactive oxygen species production by destroying the cellular antioxidant defense system and finally leads to cell demise [50,51].

### 3.7. Cytotoxicity Activity Against HeLa Cells

**Figure 6:**
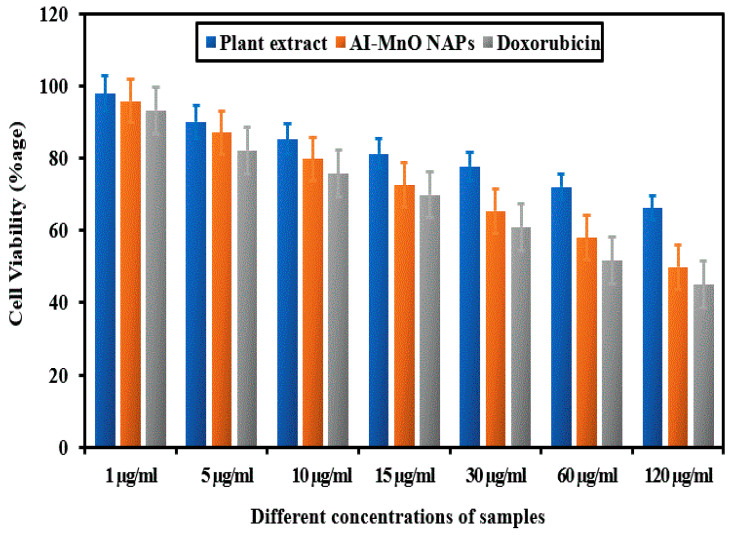
The cell viability percentage of HeLa cells treated with the plant extract and green-synthesized AI-MnO NAPs using biologically active phytomolecules of leaf extract of *Abutilon indicum* in comparison to the standard drug (*F*-value = 6659.60, *p* < 0.05).

The plant extract and green-synthesized AI-MnO NAPs were assessed for their cytotoxicity activity using MTT assay against the HeLa cancer cell line in vitro in comparison to standard anticancer drug doxorubicin. After incubation of HeLa cells with the samples and drug, a remarkable drop in cell viability was observed by increasing the concentration (1 µg/mL, 5 µg/mL, 10 µg/mL, 15 µg/mL, 30 µg/mL, 60 µg/mL, and 120 µg/mL) of tested samples (Figure 6). The maximum cytotoxicity activity was shown at 120 µg/mL concentration by standard drug and AI-MnO NAPs, respectively, because more than 50% of the HeLa cancer cells died. Dose-dependent cytotoxicity activity was observed with all the samples (plant extract, AI-MnO NAPs) and drugs, similar to antibacterial activity. Moreover, synthesized AI-MnO NAPs interestingly demonstrated a comparable cytotoxic effect on HeLa cancer cells to the standard drug. These substantial cytotoxic results were attributed to the NAPs’ physical characteristics (size, morphology, surface area) and their functionalization with the biologically active phytomolecules (polyphenols, flavonoids, terpenoids, alkaloids, etc.) of plant leaf extract because plant extract itself displayed good cytotoxicity [13,16]. Many studies revealed that *Abutilon indicum* leaf extract possesses biologically active phytomolecular functionalities [40,41]. The same results were reported for cytotoxicity of other NAPs in which higher toxicity was observed with the green-synthesized NAPs as compared with those manufactured by using other methods [10,11,12,13,14]. Hence, the synthetic process, size, shape, surface area, and functionalization with biologically active phytomolecules significantly affect the cytotoxicity of synthesized NAPs [17].

### 3.8. Biocompatibility Study

**Figure 7:**
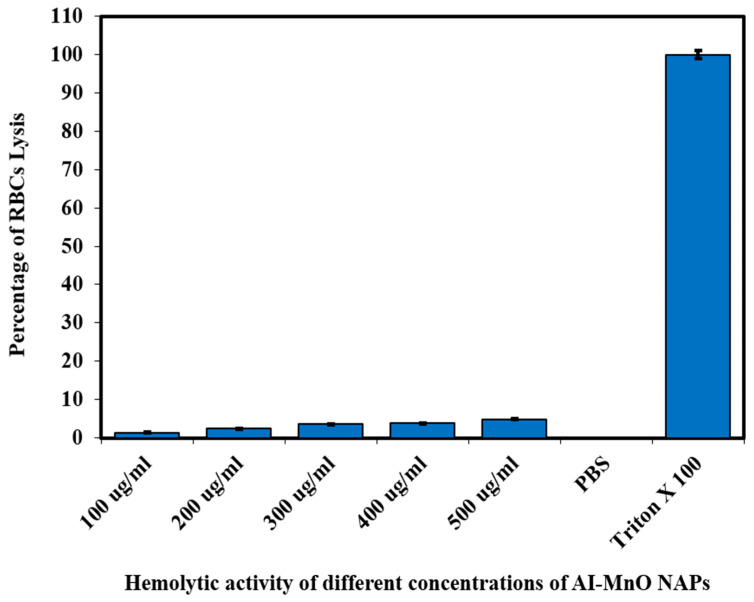
The biocompatibility of green-synthesized AI-MnO NAPs (*F*-value = 120,651.238, *p* < 0.05).

The biocompatibility of the green-synthesized AI-MnO NAPs was evaluated in vitro on red blood cells (RBCs) via the hemolytic activity. The hemolytic activity result is presented in Figure 7. The result demonstrates that triton X-100 (positive control) has the highest toxic effect while PBS (negative control) exhibited no toxicity to RBCs. On the other hand, the green synthesized AI-MnO NAPs at the concentration of 100 µg/mL displayed the least toxic effect on RBCs. The slight increase in the percentage of RBCs lysis was observed with the increasing concentration from 200 to 500 µg/mL. According to the ISO 10993-5, a material to be considered toxic or nontoxic, cell variability must be in the following order: >80% no cytotoxic, 80–60% weak cytotoxic, 60–40% moderate cytotoxic, and <40% strong cytotoxic [52]. Hence, it is interesting to note that the percentage of RBCs lysis remained less than 5% at the tested concentrations, which indicates that the green-synthesized AI-MnO NAPs are biocompatible. Our hemolytic activity results found in close agreement with the previous work reported by Khan et al. [53]. They reported the less-toxic nature of manganese oxide NAPs at the concentration of 666.44 µg/mL and considered these NAPs reliable and suitable for their biological applications [53].

### 3.9. Photocatalytic Activity against Methylene Blue (MB) Dye

**Figure 8:**
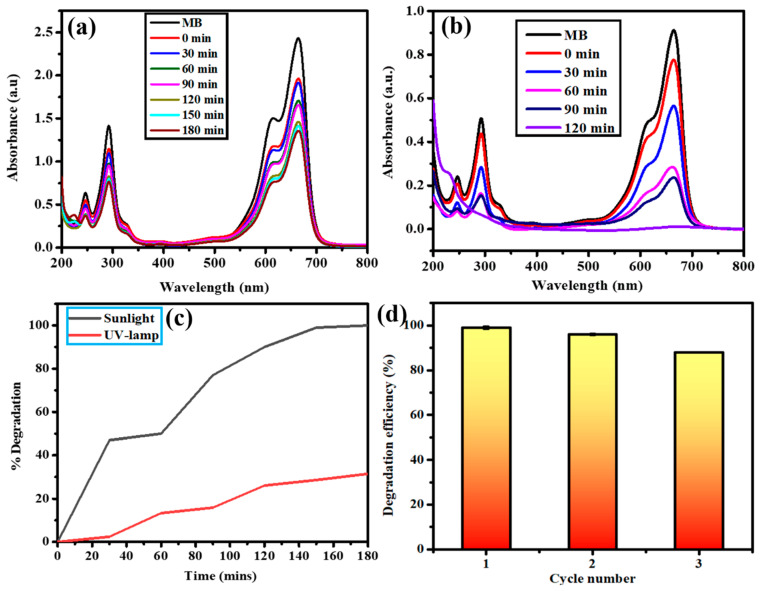
UV–vis spectra of photocatalytic degradation under (**a**) UV lamp and (**b**) sunlight. (**c**) Comparison of degradation percentage under UV lamp and sunlight. (**d**) Degradation efficiencies for three runs.

The photocatalytic disintegration ability of green-synthesized AI-MnO NAPs was determined against MB dye under the irradiation of a UV lamp and sunlight spectrum. The photocatalytic activity result of AI-MnO NAPs is presented in Figure 8. First, the MB dye was evaluated for its self-disintegration without adding synthesized photocatalyst (AI-MnO NAPs) under both spectra, and no self-degradation was found. Photocatalysis results show that green-synthesized AI-MnO NAPs significantly photocatalyzed the MB dye under the sunlight spectrum within 120 min. However, they demonstrated the least photocatalytic activity in the UV-lamp irradiation spectrum. A sufficient amount of MB dye remained unphotocatalyzed even after 180 min (Figure 8a,b).

The UV-visible spectrum shows four absorption peaks for MB dye in the range of 200–800 nm wavelength (Figure 8a,b). Absorption peaks in the visible region (612 nm and 664 nm) are attributed to nitrogen–sulfur conjugated system (chromophore); while in the UV region (246 nm and 292 nm) they are from the phenothiazine structure in MB dye [3]. Upon degradation, the intensity of these four absorption peaks was gradually reduced, which indicated the oxidative degradation of the nitrogen–sulfur conjugated system with ring-opening of phenothiazine [54]. The absorbance of MB dye solution under solar spectrum irradiation with AI-MnO NAPs approached zero after 120 min compared with UV-lamp irradiation, and the blue color of the dye was changed to colorless, indicating its complete decomposition into other products (CO_2_, H_2_O, CH_3_OH, etc.). Figure 8c demonstrates that the highest disintegration percentage of MB took place during the first 30 min and ultimately reached 100% in 120 min under sunlight spectrum, while, even after 180 min, only 31% was achieved under a UV lamp. Similar results were reported by Arasu et al. and Sheikhshoaie et al. in the degradation of MB with MnO and Mn_3_O_4_ photocatalyst under the sunlight spectrum, respectively [48,54]. 

The rate of disintegration of the synthetic dyes entirely relies on physical features such as less bandgap, highly crystalline nature, shape, large pore size, and specific surface area of the photochemical-catalyst [54,55]. In our study, green-synthesized AI-MnO NAPs demonstrated the moderate rate of photocatalytic degradation of MB. This might be because of the smaller pore size and smaller specific surface area of the AI-MnO NAPs. Further, we calculated the specific surface area theoretically by using the following equation, S = 6 × 10^3^/D_p_*ρ* (S = specific surface area, Dp = particle size, *ρ* = density of MnO NAPs—which is 5.37 g/cm^3^) [56], for the confirmation of our hypothesis. The calculated specific surface area of the synthesized AI-MnO NAPs was 13.966 m^2^/g, which was smaller and supported our hypothesis. 

In photocatalysis reactions, the recyclability and stability of the photocatalyst have significant importance [3,55]. The recyclability and stability of the green-synthesized photocatalyst were assessed. The green-synthesized photocatalyst was run for three consecutive cycles (one cycle equal to 180 min) under the same set of experimental conditions. The results demonstrated 99%, 98%, and 88% MB dye degradation efficiency with the recycled photocatalyst after each successive cycle of 180 min (Figure 7). This exhibited that the degradation efficiency of the AI-MnO NAPs in the second cycle remained statistically equivalent (*p* > 0.05) to the first cycle [57]. However, the degradation efficiency at the third cycle was observed as statistically inequivalent (*p* < 0.05) to the first and second cycles.

Based on our findings, the possible mode of action of AI-MnO NAPs (photocatalyst) to disintegrate the MB dye into other degraded products is as follows [54,55]:

AI-MnO + Sunlight → AI-MnO^+^ + e^−^,

e^−^ + O_2_ → ^•^O_2_^−^,

AI-MnO^+^ → AI-MnO + h^+^,

h^+^ + H_2_O/OH^−^ → ^•^OH,

MB dye + ^•^OH, h^+^ → CO_2_ + H_2_O (Degraded products),

MB dye + ^•^O_2_^−^ → CO_2_ + H_2_O + NH_3_ + SO_2_ (Degraded products).

**Figure 9:**
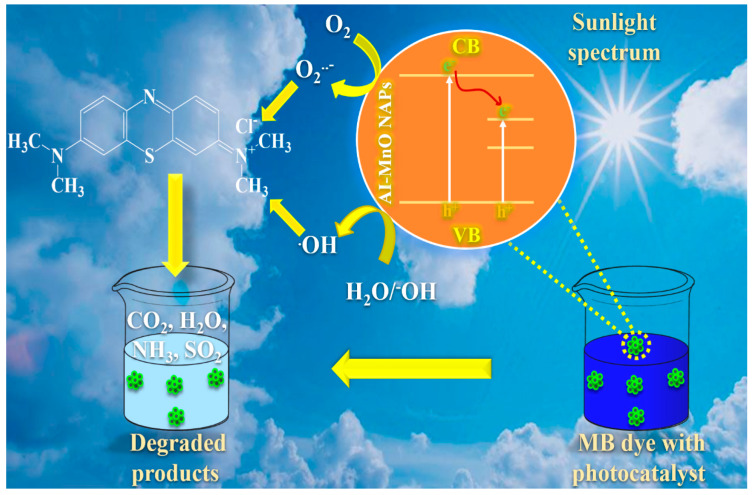
The possible mechanism of photocatalytic degradation of methylene blue (MB) dye by the green-synthesized AI-MnO NAPs using leaf extract of *Abutilon indicum*.

Accordingly, in sunlight spectrum irradiation, the electrons are generated from AI-MnO NAPs by creating a positive charge on the NAPs’ surface. The generated electrons react with the existing oxygen molecules and produce superoxide radicals (^•^O_2_^−^) [54,55]. On the other hand, holes (h^+^) produced on AI-MnO NAPs further react with the present water or hydroxyl ions H_2_O/OH^–^ and produce hydroxyl radicals (^•^OH). These generated free radical species (^•^O_2_^−^ and ^•^OH) and holes (h^+^) are highly reactive, they readily react with the MB dye and disintegrate into less harmful degraded products (CO_2_, H_2_O, CH_3_OH, NH_3_, SO_2,_ etc.) (Figure 9) [3,54,55].

### 3.10. Adsorption of Chromium(VI)

#### 3.10.1. Effect of Adsorbent Concentration

**Figure 10:**
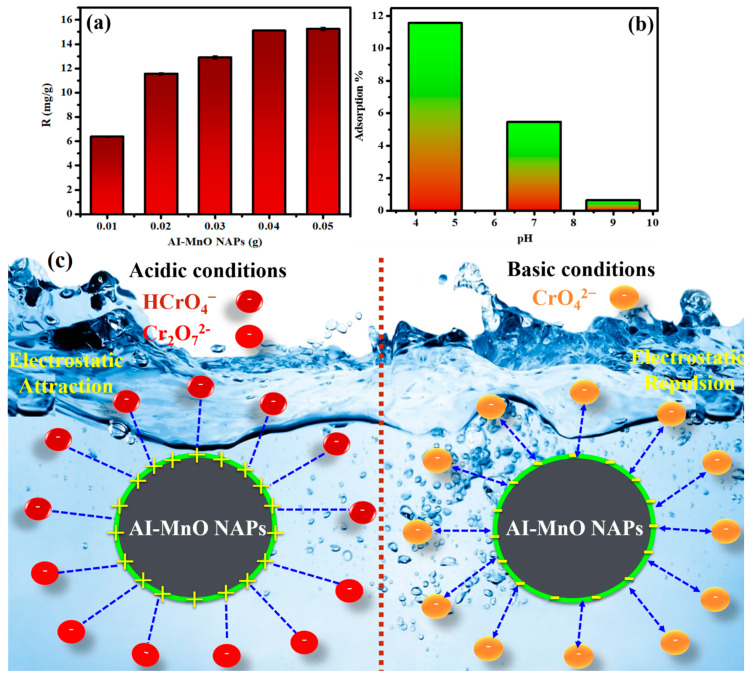
(**a**) Comparison of percentage adsorption by changing the concentration of green-synthesized AI-MnO NAPs (*F*-value = 3031.63, *p* < 0.05). (**b**) Comparison of percentage adsorption at different pH values (*F*-value = 38,499.3, *p* < 0.05). (**c**) A possible Cr(VI) adsorption mechanism on the surface of the green-synthesized AI-MnO NAPs.

The concentration of adsorbent has a significant role in the adsorption of Cr(VI) as a direct relation present between adsorbent concentration and chromium adsorption. Therefore, we have evaluated different concentrations (0.01–0.05 g) of green-synthesized AI-MnO NAPs for Cr(VI) adsorption. The adsorption capacity results demonstrated that maximum adsorption (15.25 mg/g) of Cr(VI) was observed at the concentration of 0.05 g of AI-MnO NAPs, while least (6.38 mg/g) Cr(VI) adsorption was detected at the concentration of 0.01 g of AI-MnO NAPs. The results show the concentration-dependent adsorption of Cr(VI) (Figure 10a). The similar concentration-dependent adsorption results were also reported by Du et al. by the adsorption of Cr(VI) on flower-, wire-, and sheet-like MnO_2_-deposited diatomites [58]. 

#### 3.10.2. Effect of Solution pH on Cr(VI) Adsorption

In addition to adsorbent concentration, the pH of the solution significantly affects the percentage of Cr(VI) adsorption by affecting the NAPs’ surface charge and the chemical state of chromium. This effect was investigated by varying the pH of the solution from alkaline to acidic. The results displayed the maximum Cr(VI) adsorption (11.50%) at acidic pH 4.50; on the other hand, least adsorption (0.63%) was observed at alkaline pH 9.0. Intermediate adsorption of Cr(VI) (5.46%) was anticipated at neutral pH 7.0 (Figure 10b). These results demonstrated the acidic pH-dependent Cr(VI) adsorption activity. More acidic pH produces more Cr(VI) adsorption by the green-synthesized AI-MnO NAPs (adsorbent). Moreover, the decrease in adsorption of Cr(VI) on amine-crosslinked wheat straw (adsorbent) with the increasing pH of the solution towards alkaline was also reported by Xu et al. [37].

This might be because of the presence of Cr(VI) in three different forms in solution depending on the solution pH, such as in HCrO_4_^−^ or Cr_2_O_7_^2-^ forms at highly acidic pH, and in CrO_4_^2−^ form at alkaline pH (Figure 10c) [37,58]. At higher acidic pH value 4.50, the surface of green-synthesized AI-MnO NAPs becomes more positively charged. It attracts the anionic species (HCrO_4_^−^ or Cr_2_O_7_^2-^) of Cr(VI) by the electrostatic force of attraction (Figure 10c) [37]. This can be further explained by two potential mechanisms as follows: (i) the electrostatic attraction between the positively charged surface of AI-MnO NAPs and the anionic Cr(VI) species favors the Cr(VI) adsorption in acidic media; (ii) the reduction of Cr(VI) to Cr(III) needs a huge number of protons that are only available in the acidic condition [59].

## 4. Conclusions

In this work, we have successfully synthesized AI-MnO NAPs functionalized with biologically active phytomolecules of hydroalcoholic leaf extract of *A. indicum* for the first time via a robust, economic, and eco-friendly approach. The green-synthesized NAPs were further characterized using different spectroscopic techniques, namely, XRD, SEM, EDX, EDX mapping, FTIR, and UV–visible. The green-synthesized AI-MnO NAPs demonstrated excellent antibacterial and anticancer activities against multidrug-resistant gram-positive bacteria, gram-negative bacteria, and HeLa cancer cells, which showed their potential for use in biomedical applications. Our findings also suggest that their enhanced biological activities were the result of the synergetic effect of physical characteristics (smaller size, large surface area) and adsorbed biologically active phytomolecules on their surface. Moreover, the green-synthesized AI-MnO NAPs also demonstrated good photocatalytic and adsorption activities against MB dye and Cr(VI), which displayed their effective potential to treat different organic and inorganic pollutants. Hence, the green-synthesized AI-MnO NAPs have enormous potential for applications in cosmetics, wastewater treatment, and pharmacological and nutraceutical industries. In conclusion, the *A. indicum* green synthesis is economical and beneficial for the fabrication of plant-mediated, low-cost, less toxic, and more biocompatible nanomaterials. From a future perspective, the selection of plants having biologically active phytomolecules will develop a novel platform for the green fabrication of NAPs for their biomedical applications.
